# Cardiac Baroreflex, HRV, and Statistics: An Interdisciplinary Approach in Hypertension

**DOI:** 10.3389/fphys.2019.00478

**Published:** 2019-04-30

**Authors:** Nadia Solaro, Mara Malacarne, Massimo Pagani, Daniela Lucini

**Affiliations:** ^1^Department of Statistics and Quantitative Methods, University of Milano-Bicocca, Milan, Italy; ^2^BIOMETRA Department, University of Milan, Milan, Italy

**Keywords:** neural control, non-parametric bootstrap, non-parametric inference, patterned alternatives, physiopathology, sympathetic activity, vagal activity, Winsorized correlation coefficient

## Abstract

Interests about the fine underpinnings of cardiovascular beat-by-beat variability have historical roots. Over the last decades, various aspects of the relationships between arterial pressure and heart period were taken as a proxy of the baroreflex in physiology and medicine, stimulating the interest of investigators in several interconnected scientific fields, in particular, bioengineering, neurophysiology, and clinical medicine. Studies of the overall system facilitated the emergence of a simplified negative (vagal) feedback model of the baroreflex and overshadowed the simultaneous interaction with excitatory, sympathetic positive-feedback mechanisms that would, however, better suit the model of a “paired antagonistic (parasympathetic/sympathetic) innervation of the internal organs.” From the bioengineering side, the simplicity of obtaining the series of subsequent RR intervals stimulated the analysis of beat-by-beat variations, providing a multitude of heart rate variability (HRV) indices considered as proxies of the underlying sympatho-vagal balance, and participating to the management of several important clinical conditions, such as hypertension. In this context, advanced statistical methods, used in an integrated manner and controlling for age and gender biases, might help shed new light on the relationship between cardiac baroreflex, assessed by the frequency domain index α, and the HRV indices with the varying of systolic arterial pressure (SAP) levels. The focus is also on a novel unitary Autonomic Nervous System Index (ANSI) built as a synthesis of HRV considering its three most informative proxies [RR, RR variance, and the rest-stand difference in the normalized power of low-frequency (LF) variability component]. Data from a relatively large set of healthy subjects (*n* = 1154) with a broad range of SAP [from normal (*n*_Nt_ = 778) to elevated (*n*_Ht_ = 232)] show that, e.g., α and ANSI significantly correlate overall (*r* = 0.523, *p* < 0.001), and that this correlation is lower in hypertensives (*r* = 0.444, *p* < 0.001) and higher in pre-hypertensives (*r* = 0.618, *p* < 0.001) than in normotensives (*r* = 0.5, *p* < 0.001). That suggests the existence of curvilinear “umbrella” patterns that might better describe the effects of the SAP states on the relationships between baroreflex and HRV. By a mix of robust, non-parametric and resampling statistical techniques, we give empirical support to this study hypothesis and show that the pre-hypertensive group results at the apex/bottom in most of the studied trends.

## Introduction

Since seminal studies by Sayers (1973) and Akselrod et al. (1981) a few decades ago it became clear that beat-by-beat oscillations in RR interval [usually alluded to as heart rate variability (HRV; Task Force of the European Society of Cardiology and the North American Society of Pacing, and Electrophysiology, 1996)] contain hidden information on underlying neural control mechanisms, based on the instantaneous balance between parasympathetic and sympathetic (inhibitory, excitatory) mechanisms (Malliani et al., 1991). Slowly initially, and faster subsequently, the increasing number of studies, now surpassing 23,000 in the Medline database, witness beyond doubt the growing interest on HRV as a *de facto* standard.

Even a simple cursory look at available literature, it appears that HRV may spark interest for different reasons, i.e., biological and technical, alone or combined, risking to favor debates about semantics rather than substance (Brown, 2017):

(1) First of all semantics: HRV (i.e., variability of heart rate computed as the number of beats/time in minutes) is frequently used interchangeably with RR V (i.e., variability of RR interval, in ms). RR V is taken as a proxy of PP interval (with some imprecision; Takahashi et al., 2016), and considered dependent of the dynamical interaction between the efferent vagal and sympathetic firing, combined with the humoral milieu and genetic substratum (D’Souza et al., 2014).
(2) HRV may be conceived as a proxy of the powerful beat-by-beat neural regulation of cardiovascular system in health and disease, providing a simple, non-invasive, means to estimate the changing equilibrium of the “paired antagonistic innervation” (Hess, 2014) (sympathetic and parasympathetic) governing RR interval. Contrary to historical considerations that “all autonomic nerves [are] motor” (Langley, 1921), evidence suggests that cardiac innervation can be represented by a dual pathway (sympathetic and parasympathetic) (Malliani et al., 1991) made up of mixed (efferent, i.e., motor, and afferent, i.e., sensory) nerves, subserving negative (mostly vagal) and positive (essentially sympathetic – Pagani et al., 1982; Malliani and Montano, 2002) feedback reflexes. Central structures (such as the recently highlighted Central Autonomic Network – Benarroch, 1993) coordinate and govern a number of nuclei exiting in a continuous flow of inhibitory and excitatory activity regulating the (sympatho-vagal) balance, hence eventually determining hemodynamic performance. Accordingly, any given setting of peripheral demands corresponds to a parallel distribution of arterial pressures and flows throughout the peripheral circulation. In physiological conditions at rest vagal activity prevails over sympathetic activity (White and Raven, 2014), approximately 4:1, and during activation, such as with exercise, the relationship is reversed, but even at maximal stimulation some level of vagal activity remains.
(3) HRV may be treated within a bioengineering ontology (Task Force of the European Society of Cardiology and the North American Society of Pacing, and Electrophysiology, 1996), considering the variability signal and various modalities of its management. Accordingly, mathematical manipulations may help define best ways to extract information (Haken, 1983) on the relative inhibitory and excitatory drives to the SA node, but also as a more subtle indicator of the underlying balance between positive and negative feedback circuits. Modeling and computing should not, however, be overemphasized against more attention and clinical sense (Karemaker, 1997) as suggested by a series of recent and older reviews and debates (e.g., Eckberg, 1997; Malliani et al., 1998; Paton et al., 2005; Billman, 2013; Pagani et al., 2018).
(4) In this context, advanced statistical analysis approaches combining non-parametric, robust, and resampling techniques might prove helpful to provide practical tools (e.g., graphical analysis) for easier clinical applications, or to extract unexpected relationships between variables (Lucini et al., 2018). Concurrently since initial studies, it was clear that the proxies of autonomic regulation were carrying different types of encoded information. For instance, limiting our considerations to a linear ontology, years ago we explored the use of a synthetic descriptor of the sympatho-vagal balance employing the numerical ratio between low frequency (LF) and high frequency (HF) components detected with spectral analysis of the RR variability signal (Pagani et al., 1986). Subsequently, it was also clear that amplitude (such as HRV) and frequency coding (particularly well represented by LF and HF in normalized units) provide different types of information (Pagani and Malliani, 2000). As suggested by electroneurographic recordings (Schwartz et al., 1973) and complex multivariate statistics (Lucini et al., 2018), amplitude and frequency codes should both be considered in the modeling of RR V. In this way, it is possible to reduce the number of significant proxies and minimize redundancy.
(5) Recently, we applied factor analysis in order to reduce the large number of indices that are provided by spectral analysis of RR V and found that the major part of information (82.7%) embedded in RR V is carried by three clusters of indices of homogeneous meaning (Sala et al., 2017). Factor loadings suggest the following clusters: normalized autonomic indices, absolute indices, and heart period. The introduction of a unitary Autonomic Nervous System Index (ANSI) may provide a way of further reducing information proxies (Sala et al., 2017). Notably this finding, as with all new findings, should be treated with caution.

From a clinical perspective, it is crucial to recall that HRV (particularly its time domain proxies) provides sensitive markers of prognosis in several conditions, particularly in coronary artery disease, predicting mortality in post-myocardial infarction (Huikuri and Stein, 2013).

Indeed the potential importance of assessing the short-term baroreflex control of heart rate/heart period as a means to describe clinical conditions was well established since several decades ago for hypertension (Bristow et al., 1969), heart failure (Eckberg et al., 1971), in addition to a strong predictive capacity for post-myocardial infarction mortality (La Rovere et al., 1998), even in animals (Billman et al., 1982). Implicitly these findings support the view of an integrated complex two-way (afferent/efferent) neural substratum of visceral regulation, at variance with the traditional efferent only view proposed by Langley (1921). It should also be considered that explicit acceptance of a mixed neural model of the autonomic (!) innervation could clear large fraction of the existing inconsistencies about HRV interpretation. This aspect is beyond the aim of the present study.

In the light of the above, here we aim to assess whether the application of advanced statistical tools, used in an integrated manner, might help unravel novel aspects of the (bivariate) relationships between the cardiac baroreflex and the autonomic indices (or proxies or measures) from RR V and arterial pressure variability, as initially exemplified by simple correlation. Data from a relatively large set of healthy subjects with a broad range of systolic arterial pressure (SAP, from normal to elevated) show that, in general, the frequency domain index α and the ANS proxies have significant (positive or negative) correlations. Accordingly, by a statistical data-driven approach, instead of model-based, we investigate, first, how cardiac baroreflex, as reflected by the α index, and ANS proxies [inclusive of ANSI (Sala et al., 2017)] match. We then assess how SAP levels affect these relationships, according to the study hypothesis that non-normotensive states could induce changes in the strength and significance of this kind of relationships.

We focus specifically on the role of SAP. Although arterial pressure values describe a continuum in the population, arterial hypertension definitions contemplate categories based on both systolic and diastolic thresholds, with slightly different values according to specific guidelines (Muntner et al., 2018). In this context, it is well recognized that SAP lowering (SPRINT Research Group, 2015) may be more important than diastolic blood pressure as an independent predictor of cardiovascular risk (Mourad, 2008). Systolic blood pressure also enjoys a specific role in hypertension treatment, whereby intensive lowering provides additional clinical benefit, as shown by the SPRINT Research Group (2015).

We express, from a statistical point of view, the effects of the three SAP categories (normotensive, pre-hypertensive, and hypertensive states) on the bivariate relationships between the α index and the ANS proxies as specific patterns of trends, i.e., increasing or decreasing trend as well as the so-called “umbrella” trend, which consists of concave- or convex-shape effects. To assess such effects and overcome several drawbacks inherent in the data under analysis (i.e., spurious age and gender effects, presence of subjects with outlying characteristics, improperness of the usual normality assumption), we carry out statistical analyses by combining a series of methods. Preliminarily, we set up so-called adjusted variables, i.e., the α index and the ANS proxies statistically transformed to be free of age and gender effects, in order to prevent results and conclusions of the study from potential biases caused by personal data not directly comparable (Lucini et al., 2018). On the other hand, ANSI being already free of age and gender effects by construction (Sala et al., 2017) requires no further transformation. Then, we use a robust measure of correlation computed with the Winsorizing method (Wilcox, 2012) in order to avoid potential influence of outlying subjects on the evaluation of the strength of the linear relationships under study. After that, we apply non-parametric statistical inference procedures (Hollander et al., 2014) on the Winsorized correlation (WINcorr) coefficients between the adjusted α index and adjusted ANS proxies plus ANSI to detect the presence of the hypothesized patterned effects without introducing any normality assumption. Finally, we perform all the statistical analyses in a resampling perspective according to the non-parametric bootstrap procedure (Davison and Hinkley, 1997) in order to give a more general value to the conclusions drawn. Results are displayed through convenient graphical tools that aim at providing valuable insights into the examined trends.

## Materials and Methods

Data for this observational, cross-sectional study, which is part of an ongoing series of investigations, focus on the use of autonomic indices in cardiovascular prevention. They refer to a population of 1154 ambulant subjects, who visited our outpatient Exercise Medicine Clinic for reasons varying from a health check-up to cardiovascular prevention (Lucini and Pagani, 2012) for chronic conditions, inclusive of hypertension (considering untreated, non-smokers individuals within the 17–86 years age range). The protocol of the study followed the principles of the Declaration of Helsinki and Title 45, US Code of Federal Regulations, Part 46, Protection of Human Subjects, Revised 13 November 2001, effective 13 December 2001. The project was approved by the Independent Ethics Committee of IRCCS Humanitas Clinical Institute (Rozzano, Italy). All subjects provided informed consent to participate.

### Autonomic Evaluation

Our approach to the non-invasive evaluation of autonomic regulation has recently been summarized (Lucini et al., 2018). In brief, ECG, non-invasive (Finometer, TNO, Netherlands) arterial pressure and respiratory activity (piezoelectric belt, Marazza, Italy) are acquired on a PC. Beat-by-beat data series of 5 min rest followed by 5 min upright data are analyzed off-line with dedicated software (Badilini et al., 2005). As described previously (Pagani et al., 1986), from the autoregressive spectral analysis of RR interval and arterial pressure variability, a series of indices indirectly reflecting cardiovascular autonomic modulation is derived (Table 1). The software tool (Badilini et al., 2005) labels spectral components with a center frequency of 0.03–0.14 Hz as LF, and components within the range 0.15–0.35 Hz as HF, verifying the existence of an elevated coherence between RR variability and respiration. In addition, recordings of subjects with arrhythmias or LF breathing are discarded (Lucini et al., 2017). The gain of cardiac baroreflex is also assessed by a bivariate method (α index = average of the square root of the ratio between RR interval and SA Pressure Spectral powers of the LF and HF components; Pagani et al., 1988). Finally, a unitary autonomic system index (ANSI) is derived from the three HRV most informative measures (RR, RR total power, and stand-rest difference of LF_RR_ in normalized units), as described in Sala et al. (2017). ANSI is treated as a percent ranked unitary proxy of cardiac autonomic regulation, by design free of age and gender bias. It should be pointed out that there is a still ongoing debate regarding the interpretation of individual autonomic indices, in particular LF/HF as markers of the sympathovagal balance (Billman, 2013). Of probably greater importance is the alternative view of the sympathetics and the vagi as functioning in a purely efferent system (Langley, 1921) or a sympatho-vagal dual feedback (negative and positive) organization. A summary of these aspects has recently been published (Pagani et al., 2018).

**Table 1:** Definition of the variables (ANS proxies plus ANSI) employed in the study^a^.

Vars.^b^	Units	Definition
HR	beat/min	Heart rate
RR Mean	ms	Average of RR interval from tachogram
RR TP	ms^2^	RR variance from tachogram
RR LFa	ms^2^	Absolute power (a) of LF component of RR variability (V)
RR HFa	ms^2^	Absolute power (a) of HF component of RRV
RR LFnu	nu	Normalized power (nu) of LF component of RRV
RR HFnu	nu	Normalized power (nu) of HF component of RRV
RR LF/HF	–	Ratio between absolute values of LF and HF
ΔRRLFnu	nu	Difference in LF power in nu between stand and rest
α index	ms/mmHg	Frequency domain measure of baroreflex gain
SAP	mmHg	SAP by sphygmomanometer
DAP	mmHg	Diastolic arterial pressure by sphygmomanometer
SAP Mean	mmHg	Average of systogram (i.e., SAP variability by Finometer)
SAP LFa	mmHg^2^	Absolute power of LF component of systogram
ANSI^c^		Composite index of Autonomic Nervous System computed as a synthesis of RR Mean, RR TP, and ΔRR LFnu


*^*a*^Modified from Lucini et al. (2018). ^*b*^LF components are comprised within the limits 0.03–0.14 Hz; HF components are comprised within the limits 0.14–0.45 Hz. Nu is obtained as *P*(*f*)nu = [*P*(*f*)/(RR TP-VLF)] ^∗^ 100, where *P* is the component power, *f* corresponds to either LF or HF, and VLF indicates the power of the very LF (0–0.03 Hz) component. α index = average of the square root of the ratio between RR interval and SA pressure spectral powers of the LF and HF components. ^*c*^More detailed definition in Sala et al. (2017). In brief, ANSI is computed in three stages: (1) to let the proxies be comparable in terms of magnitude, scale, and unit of measurement, and free them from age and gender effects, the percentile rank transformation is applied to RR Mean, RR TP, and ΔRRLFnu within each combination of gender and classes of age (Table 3); (2) an individual radar plot is built for each subject using the values of the transformed RR Mean, RR TP, and ΔRR LFnu. Each plot then contains a triangle with side lengths in the [0, 100] range; (3) the final ANS indicator is computed as the area of the triangles, which is subsequently transformed by percentile ranks over all the set of subjects. Ranging from 0 to 100 by construction, ANSI has an immediate clinical interpretation: the higher the value of ANSI, the better the individual autonomic condition, and vice versa, the lower the value, the worse the autonomic condition.*

### Statistics

Participants to the study, amounting to 1154 in all, were divided into the three SAP groups: normotensive (Nt), pre-hypertensive (preHt), and hypertensive (Ht), according to the definition reported in Table 2 (second column). The majority of individuals fell into the Nt group (67.4%), while the others into the Ht (20.1%) and preHt (12.5%) groups, respectively. We introduced the further subdivision of the Nt and Ht groups in the SAP intervals indicated in the last column of Table 2 in order to better meet the aims of statistical analyses, as will be described soon after.

**Table 2:** Frequency and percentage distributions of the participants to the study within the three SAP groups and further subdivision in seven SAP intervals.

SAP groups	Definition^a^	Count	Percentage	Further subdivision in SAP intervals^b^
Normotensive (Nt)	Subjects with SAP < 130 mmHg	778	67.4%	Nt1 [80,100): 84 subjs (7.3%) Nt2 [100,110): 158 subjs (13.7%) Nt3 [110,120): 282 subjs (24.4%) Nt4 [120,130): 254 subjs (22.0%)
Pre-hypertensive (preHt)	Subjects with 130 ≤ SAP < 140 mmHg	144	12.5%	preHt [130,140): 144 subjs (12.5%)
Hypertensive (Ht)	Subjects with SAP ≥ 140 mmHg	232	20.1%	Ht1 [140,160): 167 subjs (14.5%) Ht2 [160,220]: 65 subjs (5.6%)
Total		1154	100.0%	


*^*a*^Recalculated from Muntner et al. (2018). ^*b*^For Nt and Ht groups only.*

We inspected potential links between baroreflex gain and HRV using the set of the 14 ANS measures listed in Table 1, which we treated as proxies of cardiovascular autonomic modulation and SAP variability. We included, as well, ANSI, which is a composite index of ANS set up such that it is free of age and gender effects (Sala et al., 2017, and legend below Table 1). Controlling for age and gender effects was one of the main problems with which we had to cope. Age and gender are biological parameters that inevitably affect the ANS proxies and the composition of the three SAP groups, this latter shown in Table 3 within the combinations of gender and classes of age. For instance, almost 84% of Nt subjects are individuals with less than or equal to 49 years of age in both female (55.98% out of 1154) and male (44.02% out of 1154) groups. In the preHt group, this percentage reduces to 64.3% within females and 62.5% within males, while in the Ht group to 35.3% within females and 56.9% within males.

**Table 3:** Distribution of the 1154 participants to the study by gender and classes of age within the three SAP groups.

Gender				SAP groups			Total
		
				Nt	preHt	Ht	
Female	Age in class	17–30	Count	204	8	4	216
			%	41.8	14.3	3.9	33.4
		31–49	Count	204	28	32	264
			%	41.8	50.0	31.4	40.9
		50–86	Count	80	20	66	166
			%	16.4	35.7	64.7	25.7
	Total		Count	488	56	102	646
			%	100.0	100.0	100.0	100.0
Male	Age in class	17–30	Count	124	17	14	155
			%	42.8	19.3	10.8	30.5
		31–49	Count	118	38	60	216
			%	40.7	43.2	46.1	42.5
		50–86	Count	48	33	56	137
			%	16.5	37.5	43.1	27.0
	Total		Count	290	88	130	508
			%	100.0	100.0	100.0	100.0


For the same arguments extensively discussed in Lucini et al. (2018), and with the same methodology therein presented, we accomplished the comparability among the SAP groups by statistically transforming the original ANS proxies in such a way they were free of age and gender effects. In short, we fitted a two-way full ANOVA model for each ANS proxy regarded as the dependent variable, and classes of age and gender as the independent variables through their main effects and interaction. Because not affected by age and gender, the resulting ANOVA residuals (given for each proxy by the difference between observed and predicted values) were referred to as *adjusted ANS proxies*, and accordingly used in place of the original ANS proxies in all the subsequent statistical analyses.

Following Lucini et al. (2002), our study hypothesis was that potential connections, observed over the total set of subjects, between the baroreflex gain (as measured by the α index) and the other ANS proxies could differ in strength, direction, or statistical significance depending on the SAP group. Focusing specifically on the linear relation (or correlation) between the α index and the other variables listed in Table 1, we were interested in assessing whether the preHt group could represent a sort of transition state from Nt to Ht group in which the correlations between the α index and the other ANS proxies plus ANSI could even strengthen. This research question mostly arose from our experience in analyzing this type data, where frequently we had observed non-monotonic (or curvilinear) effects of SAP groups on the correlations involving the α index.

**FIGURE 1:**
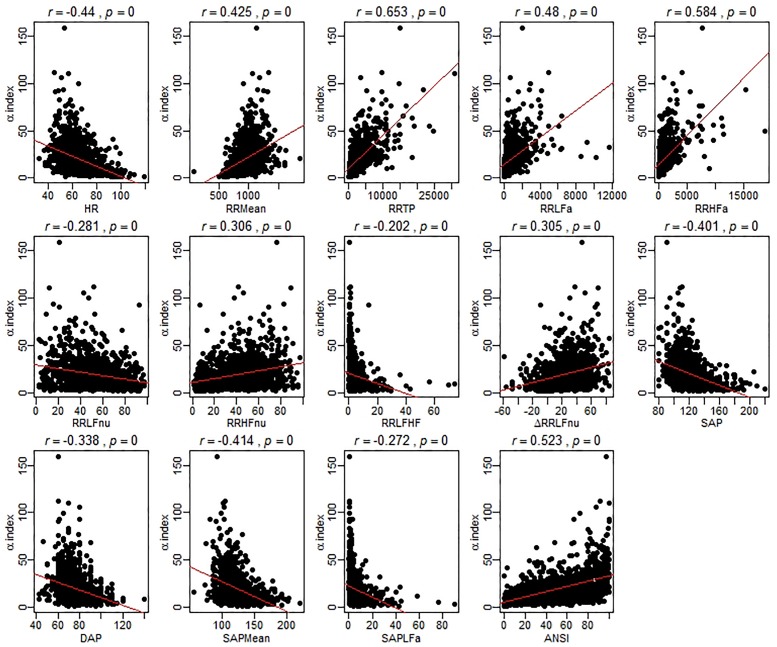
Scatter plots of the α index against the ANS proxies and ANSI over the whole set of subjects. The Pearson correlation coefficient *r* between the α index and each of the other ANS proxies plus ANSI is written above each panel, together with the *p*-value of the standard procedure for testing: *H*_0_: ρ = 0 against *H*_1_: ρ ≠ 0. In each panel, the regression line (with parameters estimated by the ordinary least-squares method) is depicted in red. It is worth stressing that these regression lines are used only as graphical references for better visualization of the spread of the point clouds, i.e., the α index is not regarded as the dependent variable of a regression model.

As a preliminary analysis illustrating the main idea, Figure 1 reports the scatter plots of the original ANS proxies and ANSI against the α index set up over the entire set of subjects, while these same graphs related to the three SAP groups are in the Supplementary Material. For each bivariate comparison involving the α index, Pearson correlation coefficients *r* and the *p*-values, obtained by the usual procedure based on the standardized normal distribution for testing the null hypothesis *H*_0_: ρ = 0 against the alternative *H*_1_: ρ ≠ 0 (at the 0.05 nominal level), are reported above each panel. All the correlation coefficients result significantly different to zero; nevertheless, as expected, they have different magnitude and sign. For example, the correlation coefficients of α and RR TP (*r* = 0.653, *p* < 0.001), and α and ANSI (*r* = 0.523, *p* < 0.001), both denote at least medium positive linear relations, while a more moderate negative correlation is observed between α and SAP Mean (*r* = -0.414, *p* < 0.001) and a weaker negative correlation between α and RR LFHF (*r* = -0.202, *p* < 0.001). Nonetheless, by performing the same kind of analysis within each SAP group, we observed that the correlations involving α might strengthen or weaken depending on the SAP groups (Supplementary Figures S1–S3 in Supplementary Material). For example, there is a medium correlation of α and ANSI overall (*r* = 0.523, *p* < 0.001) as well as in the Nt group (*r* = 0.5, *p* < 0.001), but the correlation tends to weaken in the Ht group (*r* = 0.444, *p* < 0.001) and strengthens in the preHt group (*r* = 0.618, *p* < 0.001). Again, the correlation of α and RR LFnu is weakly negative overall (*r* = -0.281, *p* < 0.001) and in the Nt group (*r* = -0.261, *p* < 0.001), but it is not significantly different from zero in the Ht group (*r* = -0.033, *p* < 0.538). All that seems then to evidence the presence of either monotonic- or curvilinear-type effects of the SAP groups on the correlations between the α index and the other variables.

The analyses performed such as in Figure 1 also opened us a critical viewpoint concerning the choice of the statistical methodology to apply. Most importantly, that kind of inspection suffers from several weakness points. First, as already observed, the original ANS proxies are affected by age and gender effects, so that a more cautionary approach would require to deal with the adjusted ANS proxies (while ANSI is already free of such effects). Second, a few anomalous values appear as isolated points in the scatter plots. These correspond to subjects having outlying characteristics on several (but not all) measures. That is a typical situation that might occur with data collected from autoregressive spectral analysis of RR variability. As known in the statistical literature, the Pearson correlation coefficient is extremely sensitive to the presence of outliers. Accordingly, one recommendation is to carry out statistical analyses by using alternative strategies. We overcame this problem by relying on robust statistical measures (Wilcox, 2012) instead of removing outlying subjects from the set of data because in this second case the total amount of the available information would have been reduced. Third, to give a more general value to the conclusions drawn on the dataset at hand, it would have been more fitting to replicate the study on additional sets of data, or alternatively, on a much broader set of data suitable to be split, e.g., at random, into a series of subsets on which replicating the analyses separately. Since the whole available dataset was large enough to meet our analysis objectives, but not large enough to be split into subsets, we decided to turn to statistical resampling techniques, such as the bootstrap (Efron, 1982; Davison and Hinkley, 1997). Finally, we preferred not to apply the classical inferential procedures based on the normality assumption, which could have been too much restrictive in our case, and carry out, instead, the analyses by a purely non-parametric approach (Hollander et al., 2014).

**FIGURE 2:**
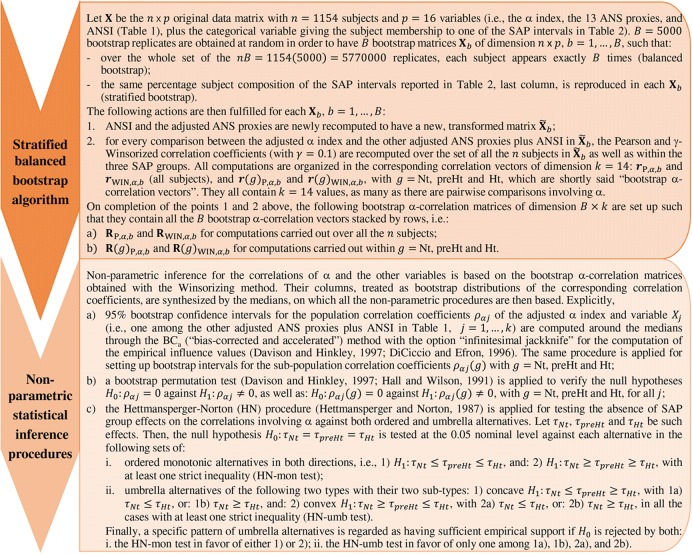
The bootstrap algorithm and the statistical methodology for non-parametric inference.

In the light of the above issues, correlations between the α index and the other variables listed in Table 1 were inspected both over the whole set of subjects and within the SAP groups by using the ANS proxies adjusted for age and gender effects and the following statistical methods (for which further methodological details are given in Figure 2):

(1) As a robust measure of correlation, we used the γ-WINcorr coefficient (Wilcox, 2012), with a proportion γ equal to 0.1. Winsorization consists of estimating the means, the variances, and the covariance involved in the Pearson correlation coefficient formula of two generic variables *X*_1_ and *X*_2_ by, first, computing their γ-th and (1 – γ)-th order quantiles and then replacing the first proportion γ and the last proportion 1 – γ of their values with these estimated quantiles. In such a way, two Winsorized distributions are obtained for *X*_1_ and *X*_2_ to which the Pearson correlation formula is applied (Wilcox, 2012);
(2) having decided to introduce no assumption for the data distribution, as a resampling technique we applied the non-parametric stratified balanced bootstrap to generate *B* = 5000 bootstrap replicates from the original data, i.e., 5000 new datasets each of size equal to *n* = 1154 subjects with *p* = 16 variables (the adjusted ANS proxies plus ANSI, and the classification variable given by the SAP group membership), which were set up such that:
  (a) by balancing, over the whole set of the *nB* bootstrap observations generated, the same subject was randomly sampled (with repetition) for exactly *B* times, but he/she might not be present in each of the *B* bootstrap replicates or might be present twice or more in any bootstrap replicate. In such a way, simulation errors were reduced considerably in comparison with the ordinary bootstrap procedure (Davison et al., 1986);
  (b) by stratification, in each of the *B* bootstrap replicates we reproduced the structure of the original data concerning the classification of subjects into the SAP groups. We had no reasonable indication for assuming a weighting schema different from the percentages computed on the original data (Table 2, fourth column). However, we had to take into account that especially the Nt group had an internal considerably heterogeneous composition [i.e., SAP ranges from 80 to 130 mmHg (excl.)] as well as the size of the three SAP groups was strongly unbalanced. Accordingly, to prevent potential distortions caused by the heterogeneous within-groups compositions and the different size of the SAP groups, we applied the bootstrap by stratifying within the SAP intervals defined in the last column of Table 2. Such intervals represent a further subdivision of the Nt and Ht groups into sub-groups of a more similar (though not equal) size that are internally more SAP homogeneous. Each bootstrap replicate was therefore randomly generated to contain *n* = 1154 (not necessarily distinct) subjects falling into the SAP intervals in the same percentages as those reported in the last column of Table 2.
  The adjusted ANS proxies and ANSI were recomputed on each of the *B* = 5000 bootstrap replicates obtained. In its turn, the WINcorr coefficient was computed on each replicate and for every comparison between the adjusted α index and the other ANS proxies plus ANSI, both over the whole set of subjects and within the SAP groups. In such a way, we obtained 5000 values of the WINcorr coefficient (i.e., a bootstrap distribution) for each type of examination and each pairwise comparison involving the α index. As a synthesis of the multitude of these bootstrap distributions, we used the median rather than the mean for reducing the influence of potential anomalous values on subsequent analysis results;
(3) non-parametric inference was drawn, both on the overall set of subjects and within the SAP groups, on the medians of the bootstrap WINcorr coefficients according to the following three approaches:
  (a) with the aim of providing plausible ranges of variation for every correlation coefficient involving the α index, non-parametric 95% bootstrap confidence intervals were computed through the BC_a_ (i.e., “bias-corrected and accelerated” intervals, given as adjusted bootstrap percentiles) method (DiCiccio and Efron, 1996; Davison and Hinkley, 1997);
  (b) to test the null hypothesis of zero correlation coefficients of the α index and the other variables, a bootstrap permutation test (Hall and Wilson, 1991; Davison and Hinkley, 1997) was applied at the 0.05 nominal significance level;
  (c) in line with the study hypothesis above described, we applied the Hettmansperger–Norton trend test (Hettmansperger and Norton, 1987) to verify the hypothesis, at the 0.05 nominal significance level, of no effect of the SAP groups on the correlation coefficients concerning the α index against the following two sets of patterned alternatives (explicitly presented in Figure 2):
    (i) the SAP groups have increasing/decreasing effects on the strength of the correlations (so-called monotonic ordered alternatives, similar to linear contrasts);
    (ii) the SAP groups have concave- or convex-shape effects on the strength of the correlations (so-called umbrella alternatives, similar to quadratic or curvilinear contrasts).

With the aim of simplifying the interpretations, we will present most of the findings obtained by the bootstrap procedure and the non-parametric inference through a synoptic figure and several graphs, such as the correlation plot and the ridgeline plot. In particular, this last graph turned out to be a powerful tool for visualization of the SAP group effects and their trend patterns, consistently with the Hettmansperger–Norton procedure.

All the statistical analyses and the pertaining routine codes were implemented in the R software, version 3.5.1 (R Core Team, 2018), along with the R libraries: “boot” for the bootstrap (Canty and Ripley, 2017); “corrplot” for the correlation plot in Figure 4 (Wei and Simko, 2017); “ggplot2” (Wickham, 2016) and “ggridges” (Wilke, 2018) for the ridgeline plots in Figure 6; “pseudorank” for the Hettmansperger–Norton test (Happ et al., 2018); and “WRS2” for computation of the WINcorr coefficients (Mair and Wilcox, 2018).

## Results

Descriptive data concerning the original ANS proxies and ANSI are given in Table 4 as means and standard deviations computed over the whole set of subjects and within the three SAP groups. A further inspection based on the box plots of the distributions of the (both original and adjusted) ANS proxies and ANSI within the SAP groups is reported in SM. As expected, RR Mean, RR TP, RR LFa, RR HFa, RR HFnu, ΔRR LFnu, and the α index present the highest mean values in the Nt group and the smallest ones in the Ht group. On the other hand, HR, SAP, DAP, SAP Mean, and SAP LFa have the highest mean values in the Ht group, and the smallest ones in the Nt group. The preHt group has the largest mean values for RR LFnu and RR LF/HF, and the Nt group the smallest ones. Regarding ANSI, it is worth observing that its mean values decrease from the Nt group to the Ht group, thus proving its sensitivity to the different ANS states observed under the various SAP conditions.

**Table 4:** Descriptive data (mean and standard deviation) of the ANS proxies and ANSI within the SAP groups and over the whole set of subjects.

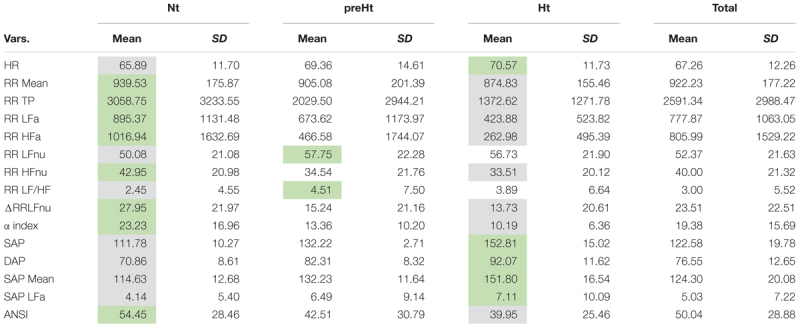

*In every comparison among the within-groups means of each variable, a green cell denotes the largest computed mean, while a gray cell denotes the smallest computed mean.*

**FIGURE 3:**
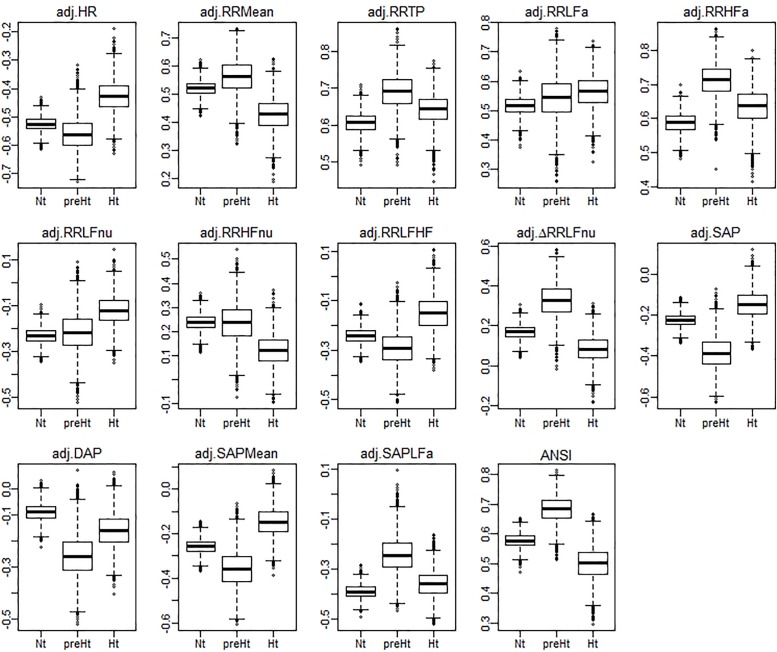
Box plots of the bootstrap distributions of the WINcorr coefficients (with γ = 0.1) of the adjusted α index and the adjusted ANS proxies along with ANSI computed within the SAP groups over the *B* = 5000 bootstrap replicates.

Regarding the bootstrap analysis, Figure 3 displays panels of box plots of the within-groups bootstrap distributions of the WINcorr coefficients (with γ = 0.1) computed for every pairwise comparison involving the adjusted α index with the other adjusted ANS proxies along with ANSI (Figure 2). At a first insight, the umbrella pattern appears in its entire evidence in line with our study hypothesis, especially in some of the panels. For example, in the first panel concerning the WINcorr coefficient between the adjusted α and HR, it can be seen a convex effect of the SAP condition on the strength of the negative correlation, i.e., the negative linear relationship between α and HR (the higher the HR values, the smaller the α values) tends to strengthen in the preHt group. On the other hand, in the last panel concerning the WINcorr coefficient between the adjusted α and ANSI, a concave effect can be clearly seen, i.e., the positive linear relationship between α and ANSI (the higher the ANSI values, the higher the α values) tends to strengthen, once again, in the preHt group.

The bootstrap within-groups WINcorr distributions in Figure 3 are used, through their medians, as empirical support to draw non-parametric inference. As a first result, Table 5 shows plausible ranges of variations, set up for both the whole set of subjects and the SAP groups, of the correlation coefficients between the adjusted α index and the other variables. These ranges are given by the non-parametric 95% bootstrap confidence intervals computed using the medians of the WINcorr coefficients. Pearson correlation coefficients of the adjusted variables computed on the original dataset are as well reported (second column, Table 5). Moreover, the cells in the first three columns of Table 5 are differently depicted according to the strength and sign of correlations (legend below Table 5). Several remarks are worth making. First, overall the medians of the WINcorr coefficients prove to be similar in both magnitude and sign to the Pearson correlation coefficients. No substantial change of interval of strength is then observed. However, winsorization has resulted in coefficients that are all, in the Nt group, or nearly all, in the whole set, slightly higher than the Pearson coefficients, while, on the other hand, in the preHt and Ht groups there is a mix of situations (i.e., some are higher, and some others are smaller than the Pearson coefficients). Moreover, by the figure reported in the legend of Table 5, it can be seen that winsorization has led, above all, to higher correlation coefficients (roughly +0.2) between α and RR LFa as well as RR HFa (in the preHt group especially), and to lower coefficients (nearly -0.1) between α and RR LF/HF as well as SAP LFa. Second, although the sign of both Pearson and WINcorr coefficients does not change across the groups, it is the magnitude that changes, especially moving from the Nt group to the Ht group. Regarding, in particular, the HRV measures RR LFnu, RR HFnu, RR LF/HF, ΔRR LFnu, along with SAP, DAP, SAP Mean, and SAP LFa, the strength of the linear relations with the α index reduces in the Ht group. Third, the bootstrap confidence intervals present fairly small widths in the whole set of subjects (0.096 on average) as well as in the Nt group (0.121 on average), thus suggesting that the strength of correlation in the various comparisons is appraised with high stability. On the other hand, the confidence intervals result wider in the preHt (0.270 on average) and Ht groups (0.240 on average), thus reflecting a greater internal heterogeneity of these two groups that is bolstered by their smaller sizes than the Nt group.

**Table 5:** Non-parametric 95% bootstrap confidence intervals for the medians of the WINcorr coefficients (*B* = 5000 bootstrap replicates, γ = 0.1) between the adjusted α index and ANS proxies plus ANSI over the whole set of subjects and within the SAP groups.

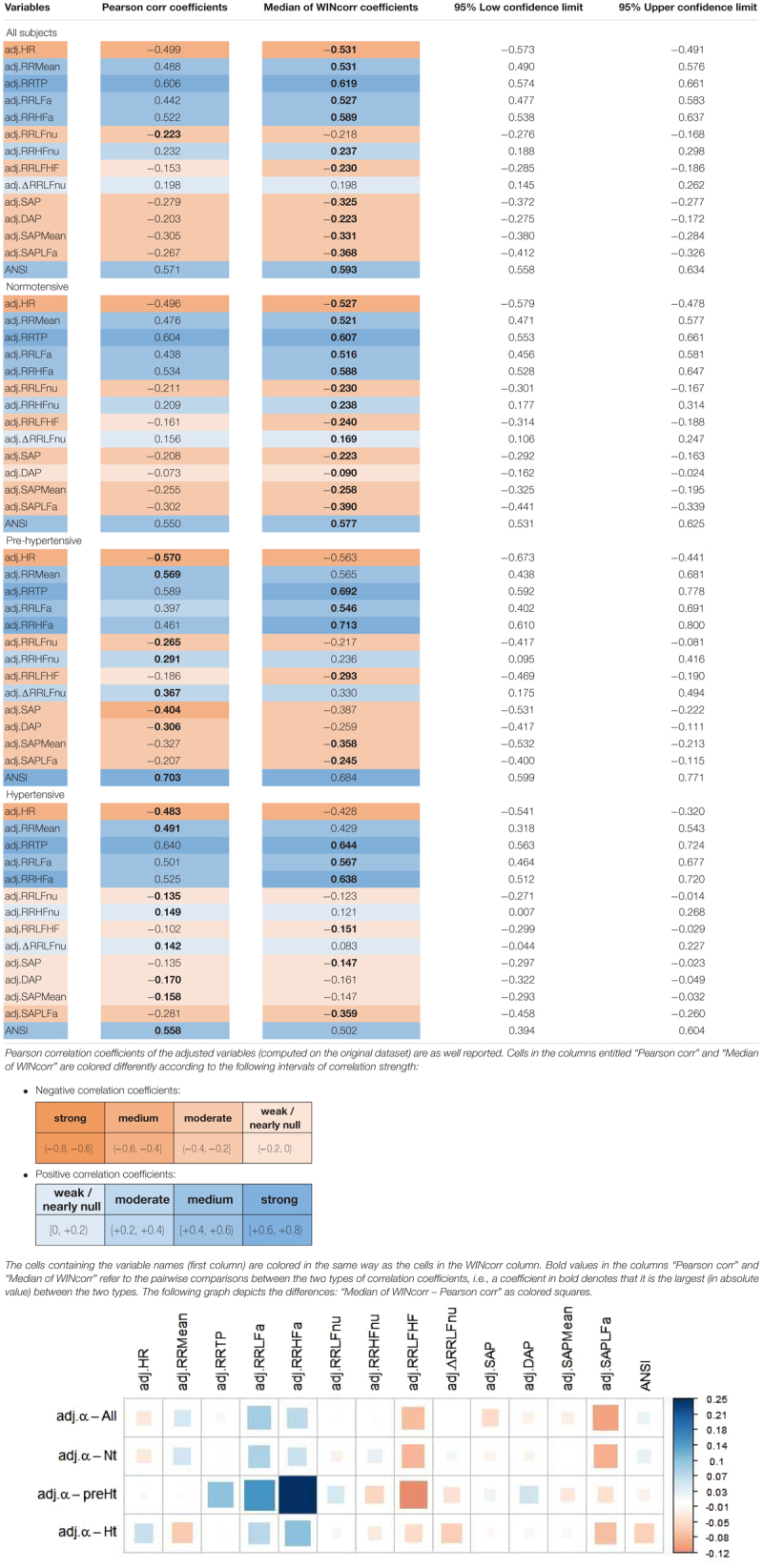

**FIGURE 4:**
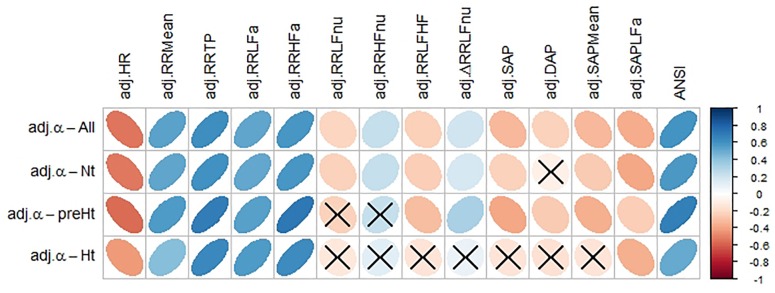
Correlation plot of the WINcorr coefficients (with γ = 0.1) between the adjusted α index and ANS proxies plus ANSI over the whole set of subjects and within the SAP groups. A cross placed on an ellipse in the cells indicates a non-significant result at the 0.05 nominal significance level achieved by the bootstrap permutation test (*B* = 5000 replicates). The corresponding *p*-values are given in the following table: 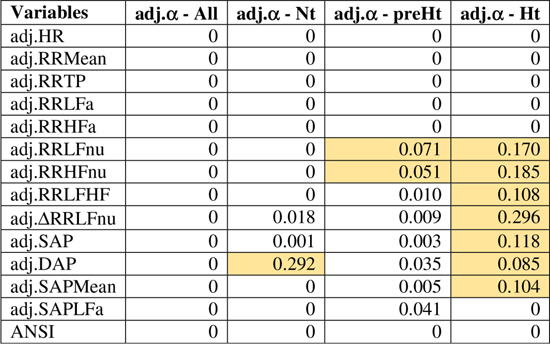 A cell is colored in light yellow in the presence of a non-significant result.

Figure 4 displays the correlation plot of the medians of the WINcorr coefficients, computed over the whole set of subjects and within the SAP groups, along with the results of the bootstrap permutation procedure for testing the hypotheses of null correlation coefficients between the α index and each of the other variables, all adjusted for age and gender effects. Corresponding *p*-values are reported in the legend. In the graph, cells containing non-significant coefficients are marked with an X symbol. As can be seen, the hypothesis of the absence of a linear relation involving the α index is accepted at the 0.05 level: in the Nt group, with DAP; in the preHt group, with RR LFnu and RR HFnu; in the Ht group, with RR LFnu, RR HFnu, RR LF/HF, ΔRR LFnu, SAP, DAP, and SAP Mean. All this seems to support our starting conjecture about the existence of SAP group effects on the pairwise relationships between α and the other considered variables.

**FIGURE 5:**
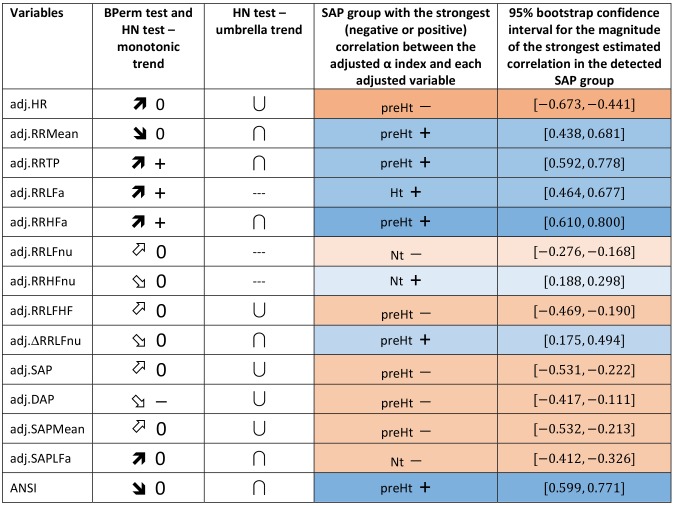
Synoptic figure summing up the results of both the bootstrap permutation (BPerm) test for null correlations and Hettmansperger–Norton (HN) test for ordered monotonic alternatives and umbrella alternatives. In all the cases in which the null hypothesis (i.e., absence of the SAP group effects) is rejected, the *p*-value is *p* <0.001. The trend of the SAP group effects on the correlation coefficients involving the adjusted α index is connoted as “increasing” or “decreasing” on the basis of the sorting of the three SAP groups in the following order: Nt as first, preHt as second, and Ht as third (like in the box plots in Figure 3). Accordingly, the symbols in the second column “BPerm test and HN test – monotonic trend” have the following meaning: 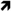 0: increasing trend from negative correlations to a nearly null correlation. 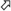 0: increasing trend from negative correlations to a not significantly different from zero correlation. 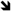 0: decreasing trend from positive correlations to a nearly null correlation. 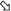 0: decreasing trend from positive correlations to a not significantly different from zero correlation. 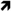 +: increasing trend from positive correlations to positive correlations. 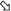 **-**: decreasing trend from a not significantly different from zero correlation to negative correlations. Moreover, the symbols in the third column “HN test – umbrella trend” have the following meaning: **∪**: convex alternative. **∩**: concave alternative. --- : absence of empirical support. Finally, in the fourth column, the SAP group with the strongest (negative or positive) correlation between the α index and each of the other variables is detected by the overall summary of all the main findings, and the pertaining 95% bootstrap confidence interval from Table 5 is reported in the last column. In particular, a blue cell with a **+** symbol reports the group where there is the strongest estimated positive correlation involving α, while an orange cell with a **-** symbol reports the group with the strongest estimated negative correlation involving α. The different shades of colors (brighter/less bright) denote confidence intervals of stronger/weaker correlations, according to the intervals of correlation strength given in the legend below Table 5.

Regarding the trend analysis, Figure 5 combines the results obtained with the bootstrap permutation test and the Hettmansperger–Norton (HN) trend test, this latter having as alternatives both monotonic ordered and umbrella effects of the SAP groups (Figure 2). Two aspects appear immediately. First, in all the considered pairwise comparisons, the HN test proves that there are at least either increasing or decreasing effects of the SAP condition on the strength of correlations between α and the other variables (Figure 5, second column). For instance, the positive correlation of α and RR TP tends to strengthen from Nt to preHt and Ht (a similar trend is observed for RR LFa and RR HFa). On the other hand, the positive correlation of α and RR HFnu tends to weaken and approach to zero from Nt to preHt and Ht. Second, in nearly all the pairwise comparisons, there is clear empirical evidence toward the presence of umbrella effects of either concave or convex shape (Figure 5, third column). For example, the negative correlation between the α index and HR, or also the positive correlation between the α index and ANSI, prove to be stronger in the preHt group than in the other two groups. The last two columns in Figure 5 sum up all the main findings concerning the detection of the SAP groups in which the strongest linear relationships involving α are observed. It is worth pointing out that in just 10 out of the total 14 pairwise comparisons the preHt group turns out to be the one in which the linear relations involving α result as the strongest ones. Specifically, in preHt, there are the strongest positive correlations between α and RR Mean, RR TP, RR HFa, ΔRR LFnu, and ANSI, respectively, and the strongest negative correlations between α and HR, RR LF/HF, SAP, DAP, and SAP Mean. On the other hand, in Nt, there are the strongest positive correlation of α and RR HFnu, and the strongest negative correlations of α and RR LFnu and SAPLFa, while in Ht, there is the strongest positive correlation of α and RR LFa. Nonetheless, saying “the strongest correlation” does not necessarily intend a correlation of high magnitude, but only that is the highest estimated correlation (in absolute value) in the comparison among the three SAP groups. Accordingly, the cells in the last two columns of Figure 5 are colored with different shades consistently with the interval of correlation strength (legend below Table 5) into which the pertaining 95% bootstrap confidence interval falls. Once again, the preHt group has a particular role because the α index proves to have a strong magnitude of positive correlation with RR HFa (95% CI: [0.610, 0.800]) and ANSI (95% CI: [0.599, 0.771]), respectively.

**FIGURE 6:**
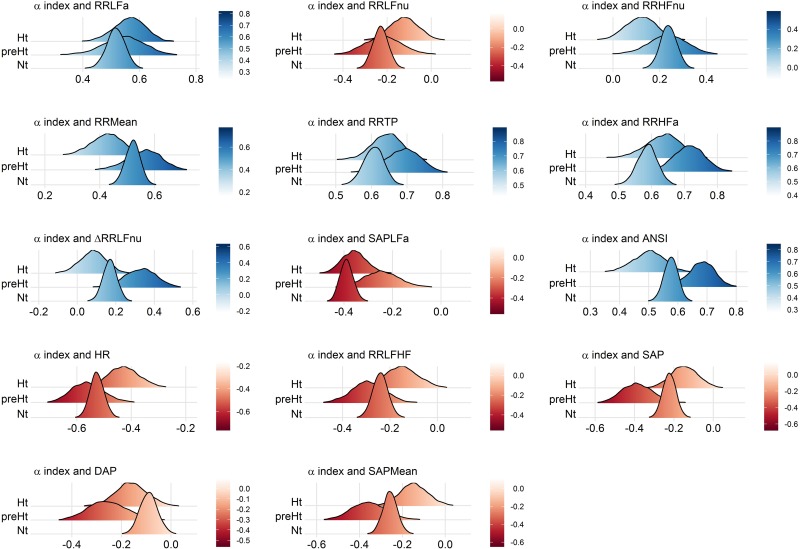
Ridgeline plots of the bootstrap distributions of the WINcorr coefficients (with γ = 0.1) of the adjusted α index and the adjusted ANS proxies plus ANSI within the SAP groups. Blue density curves denote distributions of positive correlation values, while red density curves distributions of negative correlation values. First row of panels: presence of ordered monotonic effects (increasing or decreasing) of the SAP groups. Second–third rows of panels: presence of umbrella (concave-shape) effects of the SAP groups. Fourth–fifth rows of panels: presence of umbrella (convex-shape) effects of the SAP groups.

These interpretations can be visualized better through the ridgeline plots in Figure 6, in particular by observing, in each panel, the relative position of the smoothed density curves that interpolate the bootstrap distributions of the within-groups WINcorr coefficients.

## Discussion

By using non-parametric and robust statistical procedures, combined in the perspective of a multitude of simulated replications of the study, we give empirical support to the conjecture, inspired by our empirical practice, that there exist specific pressure states in which the relationship between the cardiac baroreflex and ANS proxies can strengthen or weaken. We have turned this conjecture into practical terms by introducing the trend analysis of the SAP group effects on the correlations between the α index and each of the ANS proxies along with ANSI after adjustment for the potential biases by age and gender. We have focused on both ordered monotonic trends, i.e., increasing or decreasing effects moving from the Nt group to the Ht group, and umbrella trends, i.e., concave- or convex-shape effects concerning which the preHt group is regarded as an intermediate transition pressure condition.

Undoubtedly, a delicate issue that we had to face concerned the fact that the results found should not *strictly* depend on the adopted statistical methodology. All the more so because at present, we are not able yet to advance plausible explanations of such an observed phenomenon. In order to avoid potential straining caused by the applied statistical methods, although robust and non-parametric, we conducted an extensive preliminary study using alternative techniques to make the conclusions as far as possible untied from the specific analysis approach. In short, the results so obtained gave, in every case, empirical support to the existence of the patterned trends related to the SAP group memberships such as the ones presented.

Ultimately, the study and the statistical analyses we addressed should be more appropriately considered as a first exploratory phase toward a broader investigation that should also take into account the role of other individual characteristics (e.g., lifestyles), which we guess might affect the results found here to some extent.

### Further Considerations Concerning the Statistical Approach

As already pointed out, the statistical approach we adopted to meet the objectives of the study was designed in order to overcome several drawbacks inherent in the type of data under analysis, namely:

(a) the different age-by-gender composition of the whole set of subjects, which led us to introduce the adjusted ANS proxies (while ANSI is free of age and gender effects by construction);
(b) the presence of a small subset of outlying subjects concerning certain, but not all, variables, a problem that required us to use the WINcorr coefficient as a robust measure of the linear relationship between the α index and each of the other variables listed in Table 1;
(c) the necessity of providing a more general value to the statistical analyses without having the possibility of replicating the data collection on new sets of subjects, a fact that we overcame by turning to the bootstrap resampling technique;
(d) the improperness of the normality assumption for the bootstrap distributions of the WINcorr coefficients, for which reason we preferred to apply a non-parametric approach for both the bootstrap procedure and the inference.

Nevertheless, to ascertain which procedures or variants of the statistical methods could be the fittest ones to the data, at a preliminary stage we had to perform an extensive exploratory study and examine a range of alternative options. At the same time, this preliminary study allowed us to assess whether the main findings were as far as possible untied from the specific statistical approach used. One of the main problems was to assess the value of the proportion γ for the application of the Winsorizing method in the computation of correlation coefficients. We carried out the bootstrap procedure and all the subsequent analyses described in Figure 2 in the presence of three tentative values of the quantile order, i.e., γ = 0.05, 0.1, 0.2. Given that there were no noteworthy difference in the results, we fixed γ equal to 0.1 as a sort of “compromise value,” in order to avoid either still having a small number of outliers (γ = 0.05) or censoring the correlation coefficient distributions too much (γ = 0.2), especially in the preHt group, which is the SAP group with the smallest size (Table 2).

Another critical point was the choice of the non-parametric tests to employ against patterned alternatives. The typical distribution-free procedures adopted for testing, on the one hand, ordered monotonic and, on the other hand, umbrella alternatives are the Jonckheere–Terpstra (JT) test and the Mack–Wolfe (MW) test, respectively (Hollander et al., 2014). However, it is well-known that, in the presence of within-groups distributions with unequal variances, these tests are no more distribution-free (Hollander et al., 2014). The bootstrap within-groups distributions of the WINcorr coefficients present this problem, as it was verified on the data through the opportune procedures (i.e., the usual tests for the homogeneity of variances, results omitted). Consequently, the JT and MW test results are not sufficiently trustworthy. Among all the possible alternative procedures (Hollander et al., 2014), the choice fell on the HN test, because it is less sensitive to the inequality of variances as well as it allows specifying various patterns of trends among the alternatives in a straightforward way (Figure 2).

As a final remark, we decided to carry out the analyses between the α index and the ANS proxies according to a bivariate, rather than a multivariate, approach. We are aware that the ANS proxies are, in their turn, pairwise correlated with different strength and sign, and that a multivariate approach could have taken into account these intertwined connections at best. Nonetheless, this would have required us to use methods of synthesis of the data having as disadvantages the facts of introducing additional margins of error in the analyses as well as making the reading of the findings less clear from a clinical point of view.

### Clinical Implications and Limitations

We have shown that statistical manipulation of population data might suggest the existence of trends other than monotonic, i.e., umbrella-like, underlying the linear relationships between baroreflex gain and ANS proxies when SAP levels are taken into account. The performed statistical analyses have disclosed a peculiar role of preHt, which is positioned at the apex/bottom of a curvilinear trend in most of the examined correlations, especially between the α index and ANSI (pertaining panel in Figure 3, 6), where the correlation strengthen particularly in the preHt group (Table 5 and Figure 5).

Overall, this investigation has highlighted the existence of at least medium-strong correlations (i.e., equal to or greater than 0.4 in absolute value, Table 5) between the α index and several ANS proxies that keep in magnitude over the SAP groups. That might bear potentially important implications in the clinic, in particular, keeping in mind that the HRV proxies are extracted from the simple (ECG derived) tachogram (even if to an extent mathematically implicit). In fact, the time and resources necessary to obtain the α index [more so if using invasive arterial pressure, as originally proposed (Bristow et al., 1969)] represent a strong barrier to its introduction in the clinic, even if the clinical information provided by this measure is definitively impressive (La Rovere et al., 1998). If, on the other hand, the same (or rather similar) information is provided by simpler methods, such as by indices like HRV (Task Force of the European Society of Cardiology and the North American Society of Pacing, and Electrophysiology, 1996; La Rovere et al., 1998), the barrier in a sense could evaporate. In addition, the growing availability of simple, wearable instruments, and related SW applications is providing potentially everybody with a means to measure HRV and derived indices throughout the day and night. This possibility justifies the study of specific combinations of instruments and SW applications, ideally in a supervised network, whereby preventive strategies might take advantage of personalized markers and indices, such as ANSI (Sala et al., 2017), that profit from the analogous power of information hidden in the baroreflex as predictive tool, ready to be incorporated, by proxy (i.e., HRV indices or ANSI), into clinical routines that need, however, to be more formally tested.

Among the limitations of the study, let us point out that our findings of significant correlations fall short of cause–effect relationship that would require different approaches to be tested [e.g., moving to causal inference analyses based on structural models for causality (Pearl, 2010)]. It should also be pointed out that although significant and with medium/strong magnitude (Table 5 and Figure 5), within-groups correlation values between the α index and ANSI do not seem high enough to justify the use of ANSI to predict raw α-values. ANSI can only provide estimates of cardiac autonomic performance, as projected against a reference benchmark population (Sala et al., 2017).

## Conclusion

In conclusions, we have shown that using a combination of robust and non-parametric statistical methods, along with the bootstrap, it is possible to overcome some of the major limitations ingrained into autonomic evaluation in a clinical setting. In particular, statistical manipulation of data based on adjusted variables frees the data structure from the inherent bias related to age and gender changes. In addition, information from relatively minor study groups can be improved in quality with statistical resampling techniques such as the bootstrap, which we implemented using a non-parametric procedure to avoid assuming conjectures about the distribution of the correlation coefficients of the α index and each of the other ANS proxies. It is also important to re-emphasize (Pagani and Malliani, 2000) that we are dealing with indirect data, hence variability proxies (e.g., LF component of RR variability) cannot provide detailed information of actual, raw electrophysiological figures of nerve activity but only suggest hypothesis about (Haken, 1983) general properties of autonomic regulation, within the overall model of a dual sympatho-vagal (Hess, 2014) contrasting balance (Malliani et al., 1991).

Finally, not choosing any *a priori* model for the data structure we were able to demonstrate the validity of non-monotonic effects of the SAP states on the relationships between the α index and the ANS proxies, disclosing an umbrella-like pattern, reminiscent of the cue function of arousal (Moruzzi and Magoun, 1949). That leaves us with a crucial indication that the α index (as a proxy of baroreflex gain) is medium-strongly correlated with several indices of ANS regulation (in particular, the composite indicator ANSI), further supporting the use in a clinical setting of the simpler HRV-derived proxies, thus reducing the economic and organizational bias and potential fostering a clinical use of ANS evaluation. There are potentially practical implications in clinical management, particularly of long-term conditions where autonomic impairment might be an important issue, such as in diabetic cardiac neuropathy (Vinik and Ziegler, 2007), where traditional reflex-based models of examination have reached widespread standardization (Ewing et al., 1985). However, the introduction of novel diagnostic approaches, based on HRV and baroreflex gain, combined with advanced statistics, might facilitate the clinical assessment of graded autonomic impairment. A deeper assessment of the relationship between HRV and more complex autonomic indices, such as the baroreflex, might in addition provide a stronger and more rational basis for inferences supporting the widespread, sometimes aggressive, promotion of heart rate wearables. Furthermore, their use in a near future could also support distance-controlled, Internet-based, home-centered preventive behavioral (diet and exercise) therapies. The elevated computational power of modern wearables and smartphones, combined with the large bandwidth of Internet connections, permit to foresee applications providing personalized programs and regular support to individual patients (Wynter-Blyth and Moorthy, 2017) combining “soft” autonomic information with “hard” traditional clinical data (Editorial, 2004), avoiding to overload the health systems (Barnett, 2017).

## Ethics Statement

This study was carried out in accordance with the recommendations of the Independent Ethics Committee of IRCCS Humanitas Clinical Institute (Rozzano, Italy) with written informed consent from all subjects. All subjects gave written informed consent in accordance with the Declaration of Helsinki. The general protocol was approved by the Independent Ethics Committee of IRCCS Humanitas Clinical Institute (Rozzano, Italy).

## Author Contributions

NS, MP, and DL contributed to conception and design of the study. MM contributed to data acquisition and database organization. NS designed the statistical methodological approach, implemented the R programming codes, and performed the statistical analyses. NS and MP wrote the first draft of the manuscript. All authors wrote sections of the manuscript and contributed to manuscript revision, read, and approved the submitted version.

## Conflict of Interest Statement

The authors declare that the research was conducted in the absence of any commercial or financial relationships that could be construed as a potential conflict of interest.
