# Supplementary material for: Cardiac Baroreflex, HRV, and Statistics: An Interdisciplinary Approach in Hypertension
Source: Front Physiol. 2019 Apr 30;10:478. doi: 10.3389/fphys.2019.00478 (PMC6503090; doi:10.3389/fphys.2019.00478)
Supplement: Supplementary file 1 [file Data_Sheet_1.docx]

Supplementary Material

Cardiac baroreflex, HRV, and statistics: an interdisciplinary approach in hypertension

Nadia Solaro^1^, Mara Malacarne^2^, Massimo Pagani^2^, Daniela Lucini^2*^

^1^Department of Statistics and Quantitative Methods, University of Milano-Bicocca, Milan, Italy

^2^BIOMETRA Department, University of Milan, Milan, Italy

*** Correspondence:**Daniela Lucini
[daniela.lucini@unimi.it](mailto:daniela.lucini@unimi.it)

# Supplementary Figures and Tables

## Supplementary Figures

**Supplementary Figure S1.** Scatter plots of the ANS proxies and ANSI against α index within the normotensive group.

**
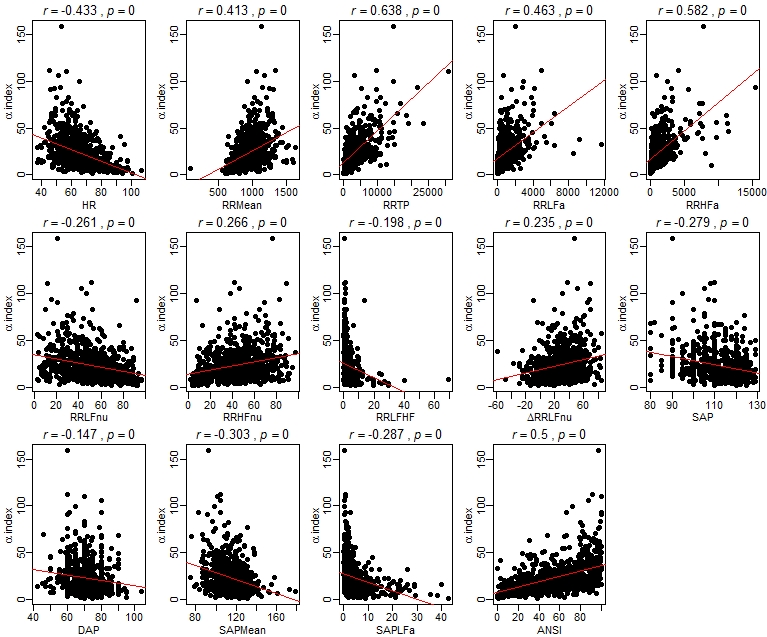
**

**Supplementary Figure S2.** Scatter plots of the ANS proxies and ANSI against α index within the pre-hypertensive group.

**
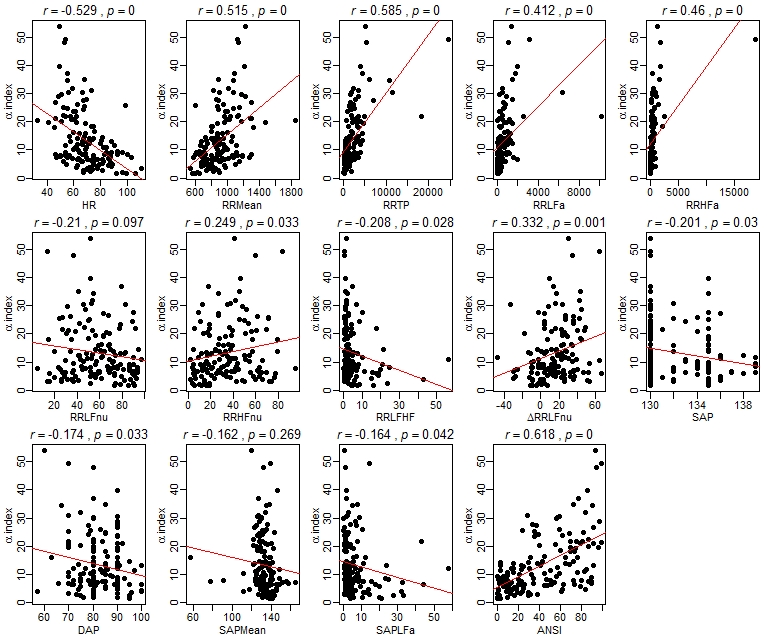
**

**Supplementary Figure S3.** Scatter plots of the ANS proxies and ANSI against α index within the hypertensive group.

**
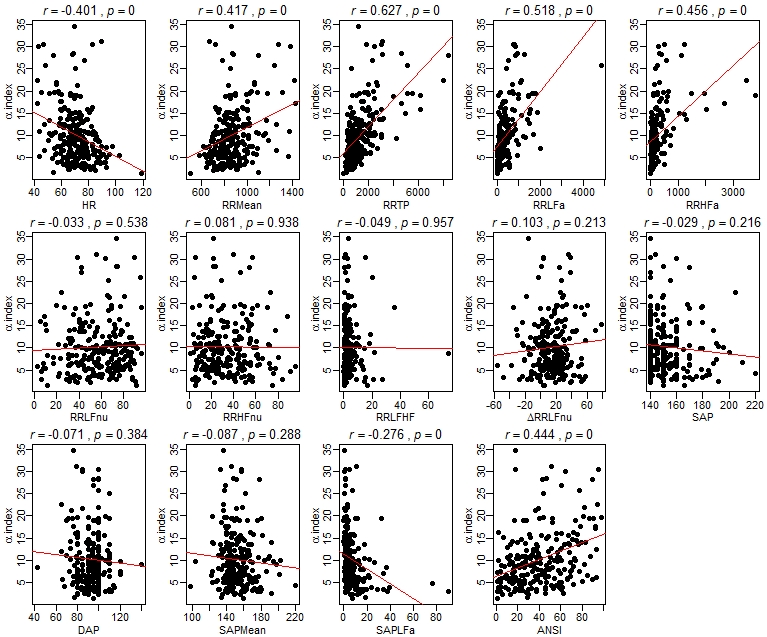
**

**Supplementary Figure S4.** Box plots of the distributions of the ANS proxies and ANSI within the SAP groups.

**
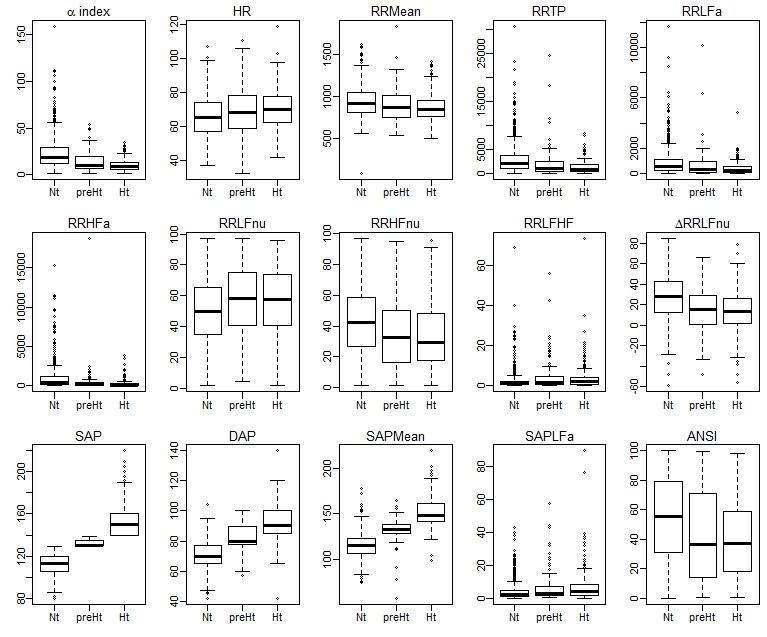
**

**Supplementary Figure S5.** Box plots of the distributions of the adjusted ANS proxies and ANSI within the SAP groups.

**
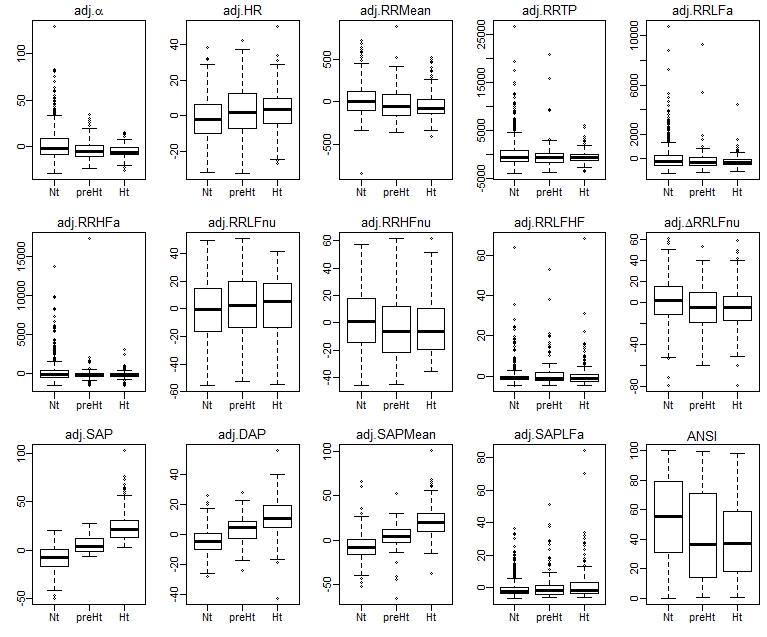
**
